# Supplementary material for: Reduction-Responsive Stearyl Alcohol-Cabazitaxel Prodrug Nanoassemblies for Cancer Chemotherapy
Source: Pharmaceutics. 2023 Jan 12;15(1):262. doi: 10.3390/pharmaceutics15010262 (PMC9864162; doi:10.3390/pharmaceutics15010262)
Supplement: Supplementary file 1 [file pharmaceutics-15-00262-s001.zip › pharmaceutics-2127600-supplementary.pdf]

Reduction-Responsive Stearyl Alcohol-Cabazitaxel Prodrug Nanoassemblies for Cancer Chemotherapy

Yuting Liu †, Xinhui Wang †, Zhe Wang, Rui Liao, Qian Qiu, Yuequan Wang * and Cong Luo *

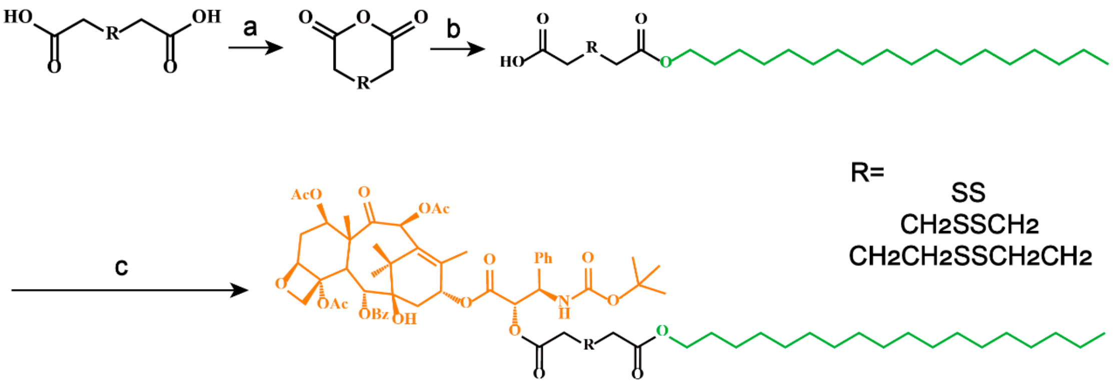

Figure S1. The synthetic route of disulfide bond-bridged CTX-SAL prodrugs. (a): acetic anhydride, 25 °C; (b): DMAP, 25 °C; (c): EDCI, HOBT, DMAP, 0 °C; CTX, 25 °C.

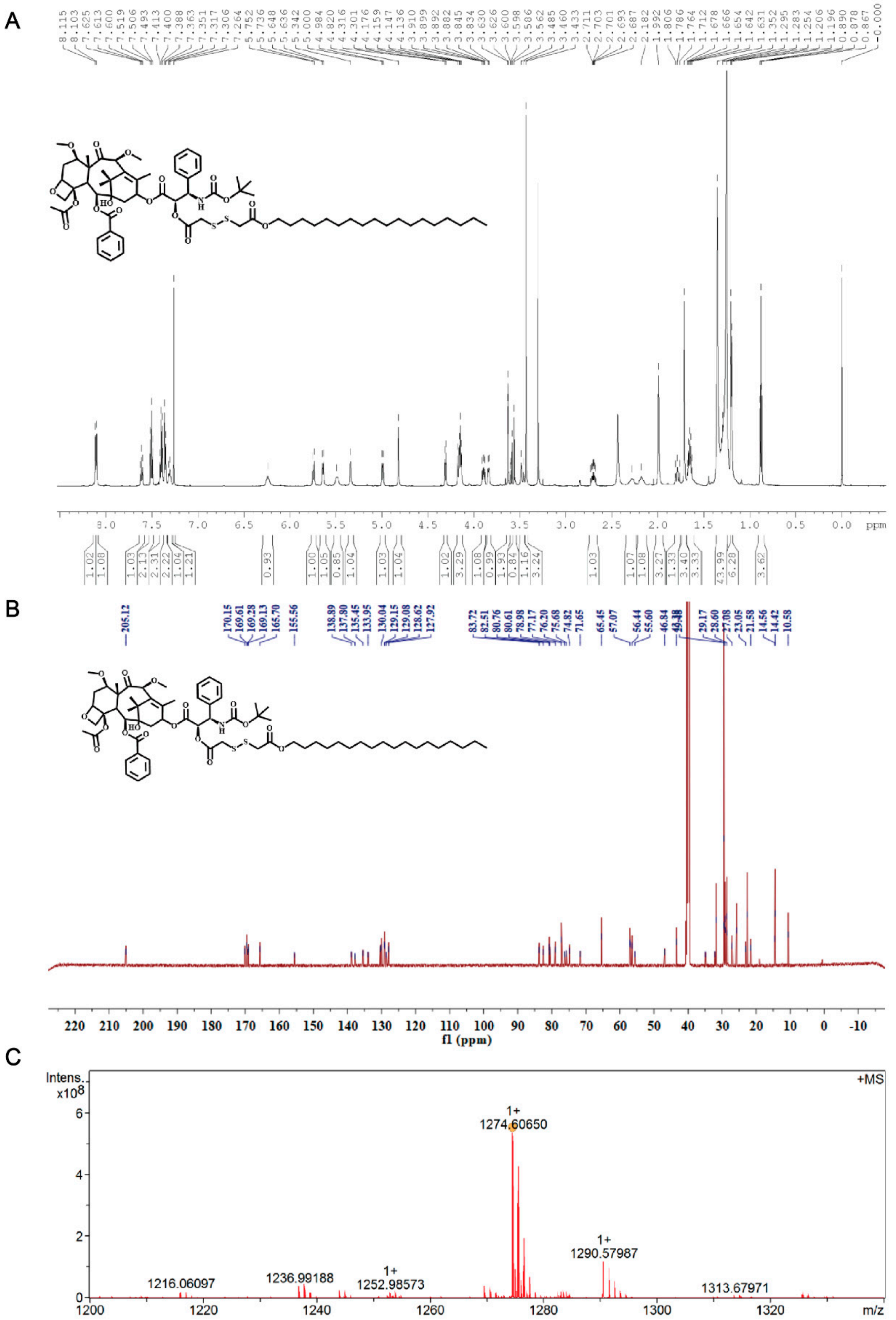

Figure S2. (A) ^{1}H NMR; (B) ^{13}C NMR and (C) MS of SAC.

^{1}H NMR (600 MHz, DMSO-d_6) δ 8.11 (s, 1H), 8.10 (s, 1H), 7.62 (t, J = 7.1 Hz, 1H), 7.52 (t, J = 8.1 Hz, 2H), 7.41 (t, J = 7.7 Hz, 2H), 7.36 – 7.35 (m, 2H), 7.31 – 7.30 (m, 1H), 7.26 (s, 1H), 6.24 (s, 1H), 5.75 (d, J = 9.7 Hz, 1H), 5.65 (d, J = 6.7 Hz, 1H), 5.49 (s, 1H), 5.34 (s, 1H), 5.00 (d, J = 9.5 Hz, 1H), 4.83 (s, 1H), 4.31 (d, J = 8.7 Hz, 1H), 4.18 – 4.14 (m, 3H), 3.91 – 3.88 (m, 1H), 3.84 (d, J

= 7.1 Hz, 1H), 3.63 (d, J = 2.7 Hz, 2H), 3.56 (s, 1H), 3.48 (s, 1H), 3.43 (s, 3H), 2.73 – 2.68 (m, 1H), 2.28 (s, 1H), 2.18 (s, 1H), 1.99 (s, 3H), 1.81 – 1.76 (m, 1H), 1.71 (s, 3H), 1.68 – 1.63 (m, 3H), 1.35 – 1.25 (m, 44H), 1.20 – 1.19 (m, 6H), 0.89 (t, J = 6.9 Hz, 3H).

^{13}C NMR (151 MHz, DMSO-d6) δ 205.12, 170.15, 169.61, 169.28, 169.13, 165.70, 155.56, 138.89, 137.80, 135.45, 133.95, 130.39, 130.04 (2C), 129.15 (2C), 129.08 (2C), 128.62, 127.92 (2C), 83.72, 82.51, 80.76, 80.61, 78.98, 77.17, 76.20, 75.68, 74.82, 71.65, 65.45 (2C), 57.07, 57.00, 56.44, 55.60, 46.84, 43.38, 34.86, 32.15, 31.76, 29.48 (10C), 29.46, 29.43, 29.40, 29.17, 29.08, 28.60, 28.53, 27.08, 25.74, 23.05, 22.56, 21.58, 14.56, 14.42, 10.58.

The m/z of SAC: $[M + Na]^+$ calculated for $C_{67}H_{97}NO_{17}S_2$, 1251.62; found 1274.61.

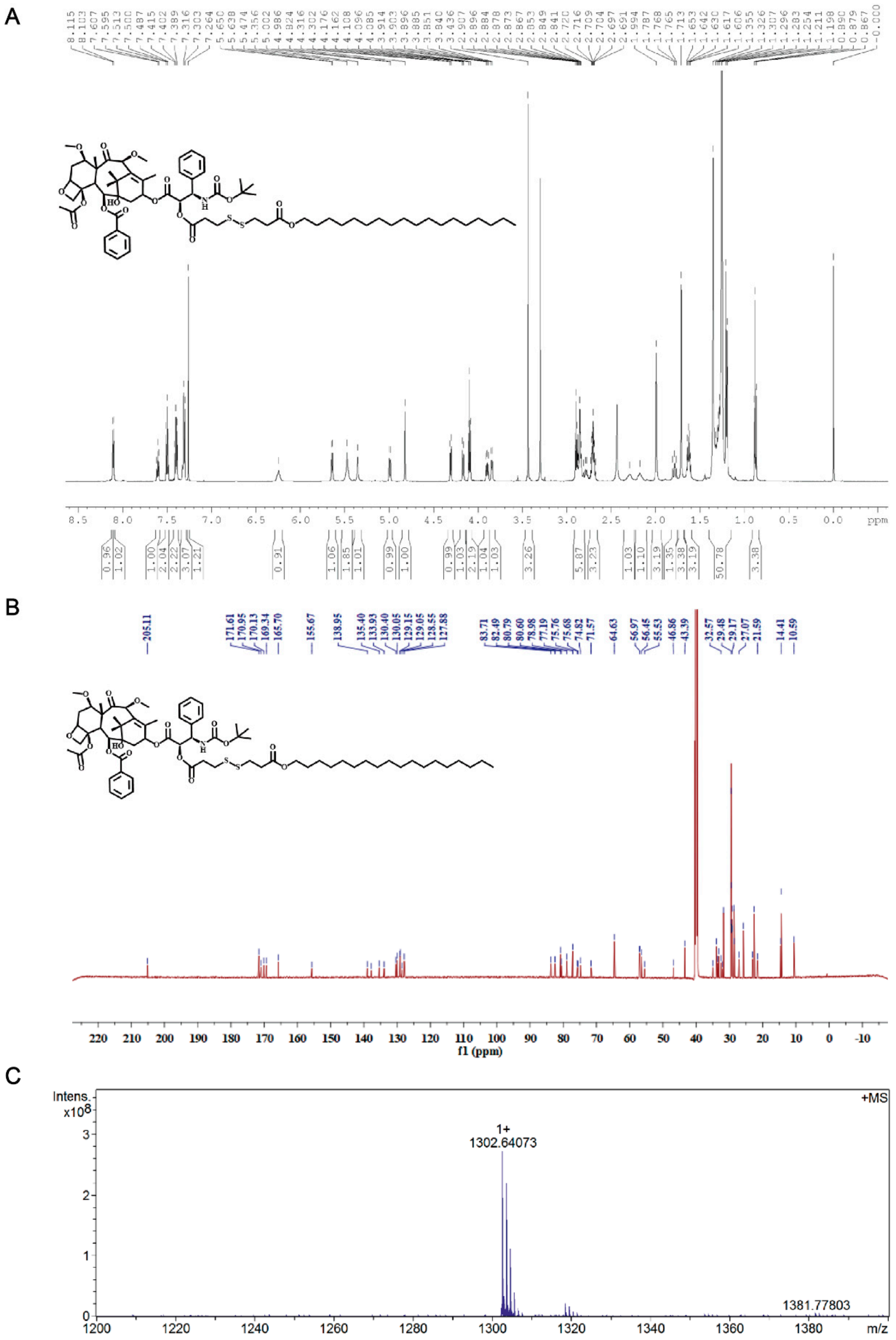

Figure S3. (A) ^{1}H NMR; (B) ^{13}C NMR and (C) MS of SBC.

^{1}H NMR (600 MHz, DMSO-d_6) δ 8.11 (s, 1H), 8.10 (s, 1H), 7.62 (t, J = 7.3 Hz, 1H), 7.51 (t, J = 7.9 Hz, 2H), 7.41 (t, J = 7.9 Hz, 2H), 7.32 – 7.30 (m, 3H), 7.26 (s, 1H), 6.25 (s, 1H), 5.65 (d, J = 6.9 Hz, 1H), 5.47 (s, 2H), 5.35 (s, 1H), 5.00 (d, J = 9.0 Hz, 1H), 4.82 (s, 1H), 4.31 (d, J = 8.6 Hz, 1H), 4.18 (d, J = 9.0 Hz, 1H), 4.10 (t, J = 6.5 Hz, 2H), 3.91 – 3.89 (m, 1H), 3.85 (d, J = 6.4 Hz, 1H), 3.43

(s, 3H), 2.90 – 2.81 (m, 6H), 2.73 – 2.68 (m, 3H), 2.29 (s, 1H), 2.18 (s, 1H), 1.99 (s, 3H), 1.81 – 1.76 (m, 1H), 1.71 (s, 3H), 1.65 – 1.61 (m, 3H), 1.35 – 1.19 (m, 50H), 0.89 (t, *J* = 7.3 Hz, 3H).
^{13}C NMR (151 MHz, DMSO-d6) δ 205.11, 171.61, 170.95, 170.13, 169.34, 165.70, 155.67, 138.95, 137.79, 135.40, 133.93, 130.40, 130.05 (2C), 129.15 (2C), 129.05 (2C), 128.55, 127.88 (2C), 83.71, 82.49, 80.79, 80.60, 78.98, 77.19, 75.76, 75.68, 74.82, 71.57, 64.63 (2C), 57.08, 56.97, 56.45, 55.53, 46.86, 43.39, 34.91, 33.92, 33.56, 33.25, 32.57, 32.16, 31.76, 29.50, 29.48 (7C), 29.47, 29.41, 29.37, 29.17, 29.08, 28.59, 28.56, 27.07, 25.80, 23.02, 22.56, 21.59, 14.55, 14.41, 10.59.
The m/z of SBC: $[M + Na]^+$ calculated for $C_{69}H_{101}NO_{17}S_2$, 1279.65; found 1302.64.

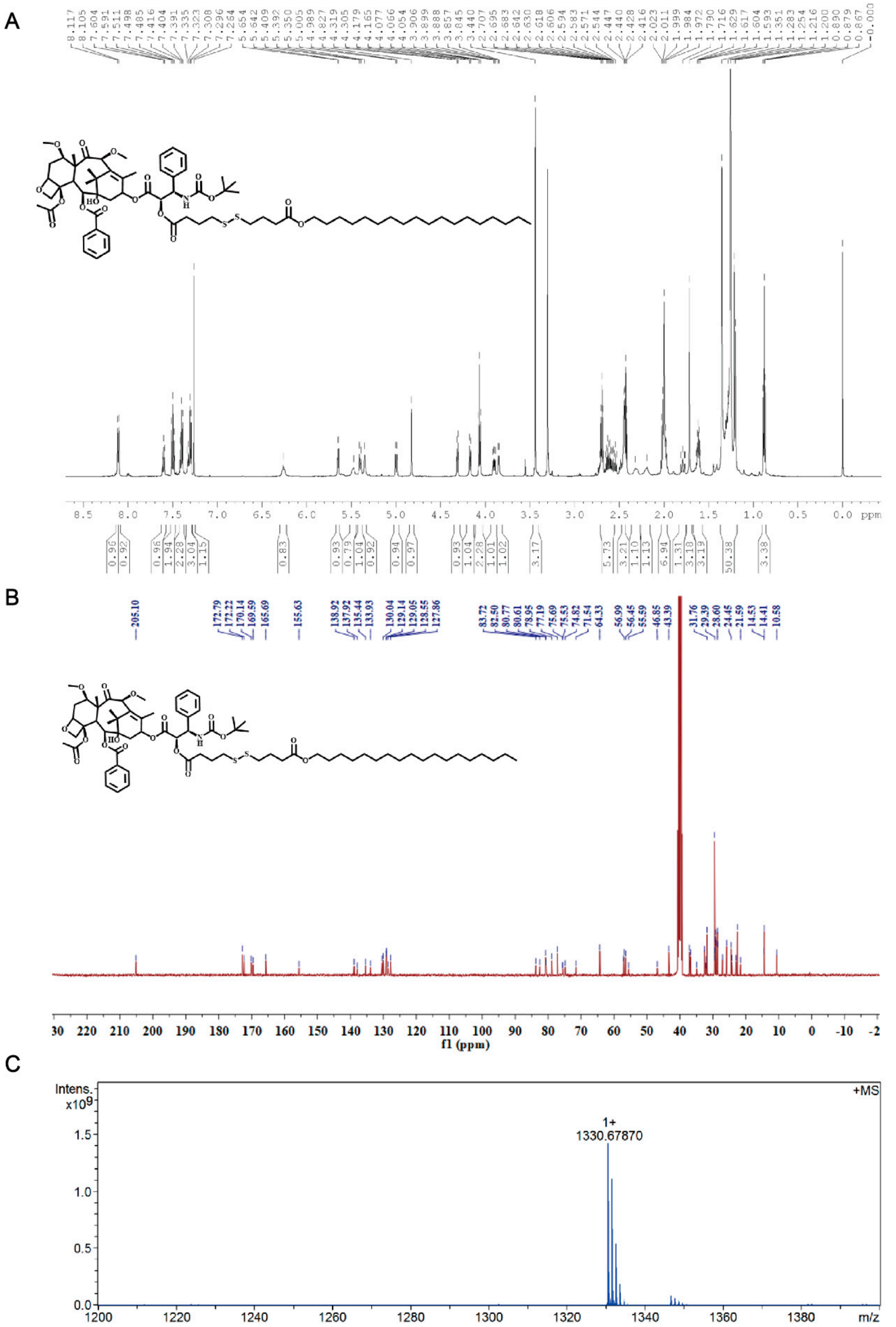

Figure S4. (A) ^{1}H NMR; (B) ^{13}C NMR and (C) MS of SGC.

^{1}H NMR (600 MHz, DMSO-d_6) δ 8.11 (s, 1H), 8.10 (s, 1H), 7.62 (t, J = 7.1 Hz, 1H), 7.51 (t, J = 8.1 Hz, 2H), 7.42 (t, J = 7.3 Hz, 2H), 7.34 – 7.30 (m, 3H), 7.26 (s, 1H), 6.26 (s, 1H), 5.65 (d, J = 6.7 Hz, 1H), 5.47 (s, 1H), 5.40 (d, J = 10.5 Hz, 1H), 5.35 (s, 1H), 5.01 (d, J = 9.6 Hz, 1H), 4.82 (s, 1H),

4.32 (d, J = 8.6 Hz, 1H), 4.18 (d, J = 8.8 Hz, 1H), 4.08 (t, J = 6.9 Hz, 2H), 3.92 – 3.89 (m, 1H), 3.85 (d, J = 6.5 Hz, 1H), 3.44 (s, 3H), 2.72 – 2.53 (m, 6H), 2.45 – 2.42 (m, 3H), 2.31 (s, 1H), 2.19 (s, 1H), 2.02 – 1.97 (m, 7H), 1.81 – 1.77 (m, 1H), 1.71 (s, 3H), 1.64 – 1.59 (m, 3H), 1.35 – 1.20 (m, 50H), 0.89 (t, J = 6.9 Hz, 3H).

^{13}C NMR (101 MHz, DMSO) δ 205.10, 172.79, 172.22, 170.14, 169.59, 165.69, 155.63, 138.92, 137.92, 135.44, 133.93, 130.40, 130.04 (2C), 129.14 (2C), 129.05 (2C), 128.55, 127.86 (2C), 83.72, 82.50, 80.77, 80.61, 78.95, 77.19, 75.69, 75.53, 74.82, 71.54, 64.33 (2C), 57.08, 56.99, 56.45, 55.59, 46.85, 43.39, 37.13, 36.77, 34.91, 32.49, 32.16, 32.08, 31.76, 29.50, 29.48 (7C), 29.45, 29.39, 29.37, 29.17, 29.07, 28.60, 28.57, 27.07, 25.83, 24.45, 24.22, 22.99, 22.56, 21.59, 14.53, 14.41, 10.58.

The m/z of SGC: $[M + Na]^+$ calculated for $C_{71}H_{105}NO_{17}S_2$, 1307.68; found 1330.68.

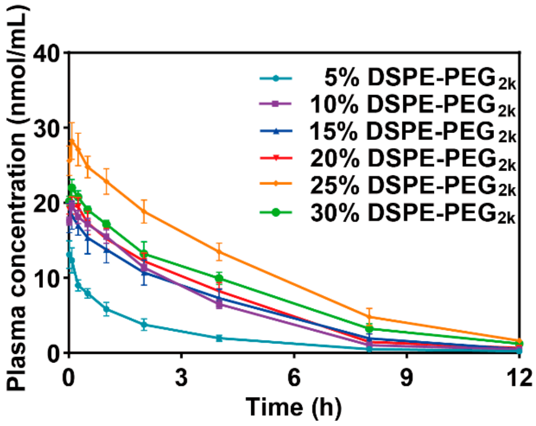

Figure S5. Molar concentration-time curves of SBC NPs with different amount of PEG (w/w).

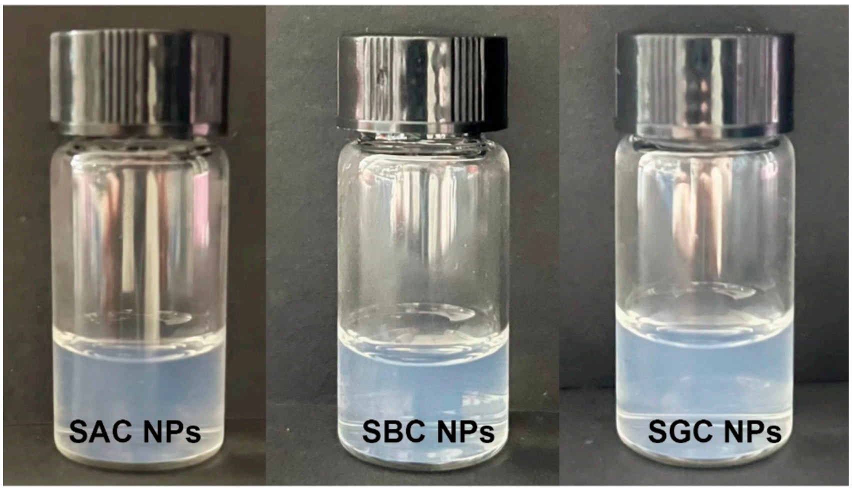

Figure S6. The appearance of three nanoassemblies at concentrations of 1 mg/mL.

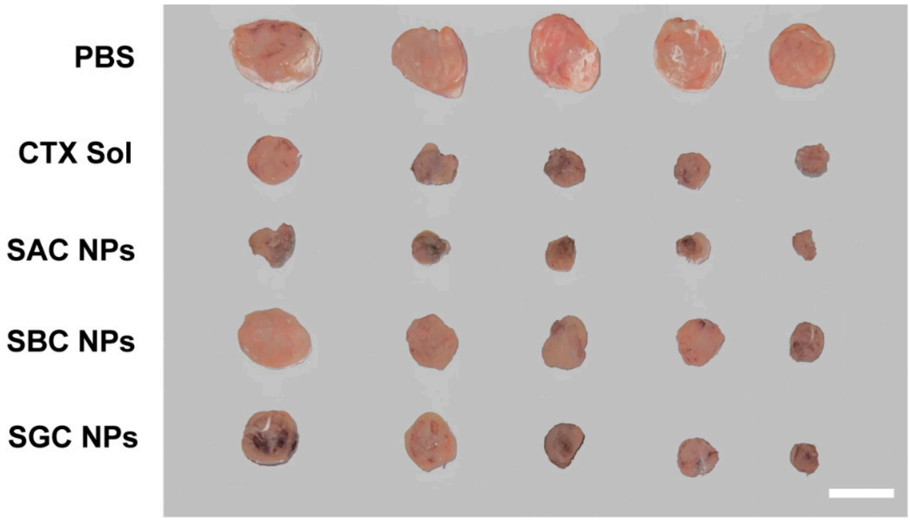

Figure S7. *In vivo* antitumor activity of prodrug nanoassemblies against 4T1 xenograft tumors (n=5). Image of tumors after last treatment. (Scale bar represents 1 cm)

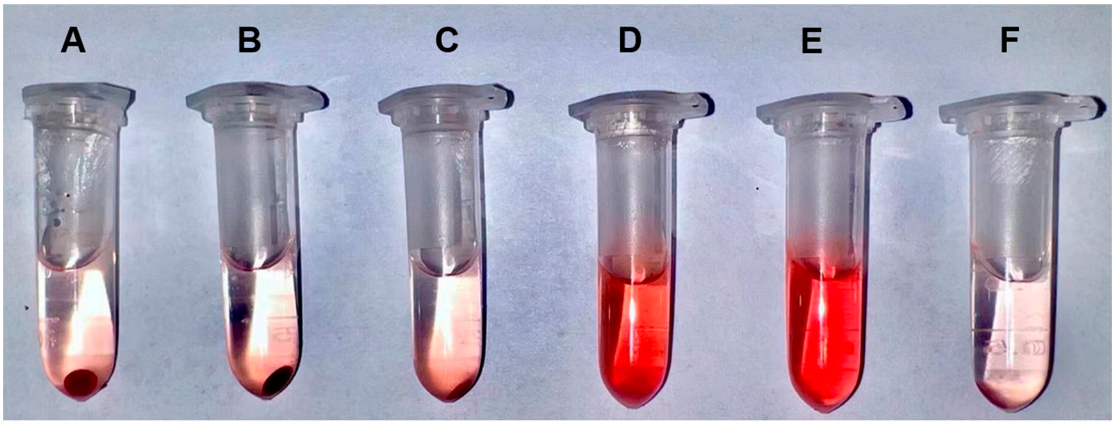

Figure S8. Hemolysis of SAC NPs (A), SBC NPs (B), SGC NPs (C), CTX solution (D), positive control (E) and negative control (F) at 4 h.

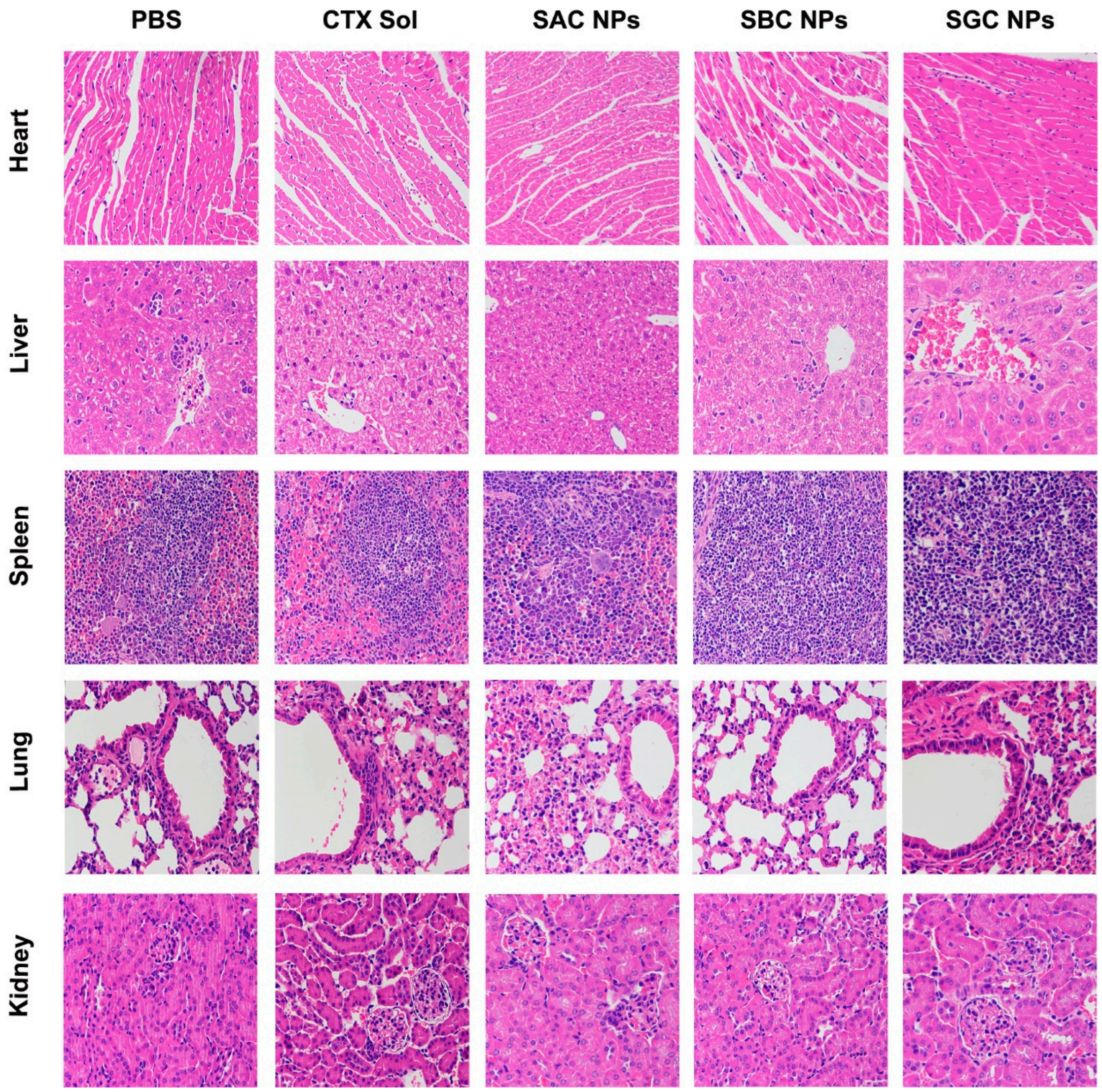

Figure S9. Representative H&E-stained of major organs (heart, liver, spleen, lung and kidney) after the last treatments.

Table S1. Characterization of PEGylated prodrug nanoassemblies. Data were presented as the mean ± SD (n=3).

Formulations	Size (nm)	Zeta potential (mV)	DL (w/w, %)
**SAC NPs**	70.11 ± 0.81	-16.40 ± 2.11	50.09
**SBC NPs**	82.96 ± 1.31	-18.63 ± 0.31	48.99
**SGC NPs**	91.77 ± 0.51	-21.60 ± 2.06	47.94

Table S2. IC_{50} (ng/mL) of CTX solution and prodrug NPs against 4T1 and 3T3 cells. Data were presented as the mean ± SD (n=3).

Formulations	4T1	3T3
**CTX Sol**	2.711	96.51
**SAC NPs**	80.43	1975

**SBC NPs**	325.2	12287
**SGC NPs**	147.2	21590

Table S3. The tumor-selective index (SI) of CTX solution and prodrug nanoassemblies between normal cells and tumor cells.

**Cell lines**	**CTX**	**SAC NPs**	**SBC NPs**	**SGC NPs**
**4T1 cells**	35.59	60.15	37.78	146.67

Table S4. Pharmacokinetic parameters of CTX solution and prodrug nanoassemblies. Data were presented as mean ± SD (n=5).

**Formulations**	**AUC_{0-24h} a)**	**$t_{1/2}$ b)**	**MRT c)**
**CTX Sol**	1.34 ± 0.41	1.10 ± 0.77	0.98 ± 0.35
**SAC NPs**	57.66 ± 4.84	2.73 ± 1.33	2.93 ± 0.43
**SBC NPs**	26.48 ± 2.47	2.19 ± 1.13	2.24 ± 0.16
**SGC NPs**	14.30 ± 1.88	2.22 ± 0.40	2.39 ± 0.33

a) Area under the plasma concentration-time curve (ug/L*h). b) Half-life (h). c) Mean residence time.

Table S5. The hemolysis rate of CTX solution, SAC NPs, SBC NPs and SGC NPs at 2h, 4h and 6h.

**Formulations**	**2 h**	**4 h**	6 h
**CTX Sol**	60.53 ± 3.78	63.70 ± 2.35	71.53 ± 3.31
**SAC NPs**	1.26 ± 0.12	1.90 ± 0.15	6.13 ± 0.25
**SBC NPs**	-0.31 ± 0.15	3.28 ± 0.15	13.35 ± 0.17
**SGC NPs**	0.58 ± 0.06	3.92 ± 0.21	5.87 ± 0.40
